# Surfactin from *Bacillus subtilis* enhances immune response and contributes to the maintenance of intestinal microbial homeostasis

**DOI:** 10.1128/spectrum.00918-24

**Published:** 2024-10-29

**Authors:** Junpeng Jia, Mei Fu, Wenxin Ji, Ningna Xiong, Peng Chen, Jian Lin, Qian Yang

**Affiliations:** 1 MOE Joint International Research Laboratory of Animal Health and Food Safety, College of Veterinary Medicine, Nanjing Agricultural University, Nanjing, Jiangsu, China; 2 Beijing Enhalor International Tech Co., Ltd., Beijing, China; University of Warwick, Coventry, United Kingdom

**Keywords:** surfactin, immune, mucosal protein, gut microbial

## Abstract

Surfactin, a lipopeptide biosurfactant produced by *Bacillus* spp., has emerged as a promising bioactive compound due to its potent inhibitory effects on bacterial and viral pathogens. This showcases its potential as a non-antibiotic strategy for managing infectious diseases. Our investigation reveals that surfactin administration significantly promotes weight gain and improves immune organ indices in mice, reflecting enhanced immunity and gut health. Surfactin augments phagocytic function in peritoneal macrophages and boosts proliferative responses in splenic lymphocytes post-chicken red blood cell immunization. Furthermore, it increases intestinal villi height, indicative of superior nutrient absorption. It elevates mucin secretion and expression of intestinal mucosal proteins, such as secretory IgA, Muc1, and Muc2, and tight junction proteins claudin-1, occludin, and Zo-1 in the jejunum and colon. Crucially, surfactin modifies the gut microbiota composition by reducing *Escherichia coli* populations and ameliorating cyclophosphamide-induced gut dysbiosis. Our data suggest that oral surfactin could be a valuable therapeutic modality to alleviate immune suppression and gut damage, proposing a new pathway for immunomodulatory treatment.

**IMPORTANCE:**

The potential of surfactin as a microbial surfactant extends beyond its surfactant properties, impacting immune regulation and gut health. As the need for alternatives to traditional antibiotics continues to grow, surfactin’s ability to enhance host defense mechanisms against common pathogens without directly targeting them with antibiotics offers a strategic advantage. Understanding how surfactin shapes the immune landscape and the gut microbiome can inform innovative interventions against immunosuppression and intestinal impairment, particularly in contexts such as cyclophosphamide-induced toxicity.

## INTRODUCTION

The intestine, one of the largest immune organs in the body (1), houses a sophisticated immune system. Its mucosal layer is abundant in lymphoid tissues, encompassing gut-associated lymphoid tissue and Peyer’s patches (2), which play a pivotal role in defending against pathogen invasion and maintaining immune homeostasis (3–5). However, immune suppression can result in dysregulation of intestinal immunity (6), which might trigger gastrointestinal inflammation and associated diseases, potentially increasing mortality rates in severe cases (7–9). Consequently, the development and application of safe immunomodulatory agents hold immense significance. Such interventions are crucial for preventing or mitigating intestinal disorders induced by immune suppression, enhancing host immunity, and promoting overall health maintenance.

Surfactin, a potent lipopeptide, has garnered significant attention for its broad-spectrum antimicrobial and antiviral properties (10). First, in poultry farming, it has demonstrated effectiveness against avian pathogenic *Escherichia coli*, offering a promising alternative in the fight against antibiotic resistance (11). Second, surfactin has shown notable antiviral activity in swine health, particularly against the porcine epidemic diarrhea virus, underscoring its potential for broader application in pig health management (12). Additionally, studies in mice have revealed that surfactin treatment can reverse symptoms of dextran sulfate sodium (DSS)-induced colitis, enhancing intestinal barrier integrity and reducing inflammation, further supporting its potential therapeutic benefits (13). However, the underlying mechanism of surfactin’s effect on immune functionality in diseases associated with immune suppression remains poorly understood and needs further investigation.

Cyclophosphamide (CTX), classified as a nitrogen mustard derivative, functions as an alkylating agent and is extensively employed in oncological therapeutics for various cancer types (14). Despite its potent cytotoxicity against neoplastic cells, cyclophosphamide can harm normal cells, leading to several side effects, including immunosuppression. This drug achieves immunosuppression by inhibiting the activity of T and B lymphocytes within the immune system and inducing lymphocyte apoptosis, thereby reducing immune cells’ quantity and functionality (6). Consequently, cyclophosphamide is frequently utilized to develop immunosuppressive models in mice and other animal subjects (15). Furthermore, this compound has been observed to compromise the integrity of the intestinal mucosal barrier, subsequently causing disruptions in gut microbiota composition and directly impacting host health (7, 8, 16).

In this study, we are investigating the influence of *Bacillus subtilis* (*B.s*) surfactin on immunomodulation in mice, specifically utilizing a cyclophosphamide-induced model of immunosuppression. We try to elucidate the advantageous role of surfactin in alleviating immunosuppression within this model. Furthermore, we aim to unravel the underlying mechanisms through which surfactin effects immune regulation and the intestinal microbiota. This investigation seeks to enhance our comprehension of surfactin’s immunomodulatory capabilities and sheds light on its potential therapeutic applications in conditions characterized by compromised immunity.

## RESULTS

### The immunomodulatory effects of surfactin in murine models

**Fig 1:**
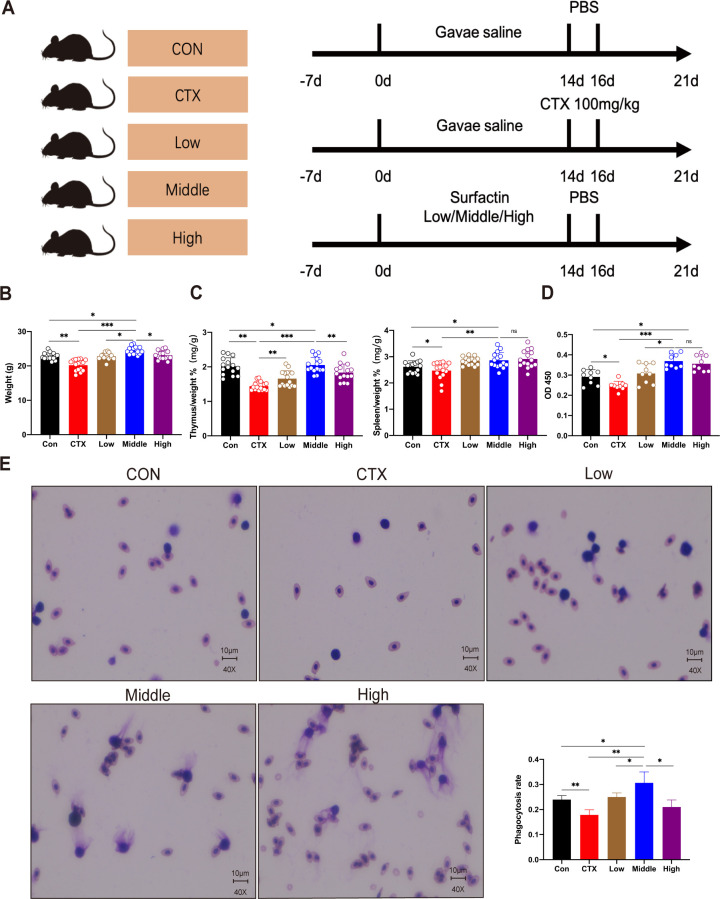
The immunomodulatory effects of surfactin in murine models. (A) Animal experiment design schematic. (**B**)The mice’s body weights in the five groups from day 0 to day 21 are plotted and presented as mean (SE) (*n* = 15/group). (**C**)Five experimental groups of mice had thymic index and spleen index (*n* = 15/group). (**D**)Effect of ConA-induced transformed function of mice splenic lymphocytes (*n* = 9/group). (**E**)Representative images of phagocytosis of erythrocytes from chicken blood by mononuclear macrophages isolated from the peritoneum of mice, following Giemsa staining (*n* = 6/group) and phagocytosis rate of mice peritoneal mononuclear macrophages (*n* = 6/group). Data represent the mean ± SD of two or three independent experiments; comparisons performed with *t*-tests (two groups). *P < 0.05, **P < 0.01, ***P < 0.001,ns no statistical significance.

In this study, we assessed the impact of cyclophosphamide and various doses of surfactin on a mouse model by evaluating physical characteristics, body weight, and organ indices (Fig. 1A). Initially, all mice exhibited normal appearance with no significant differences in weight among the groups (*P* > 0.05). Following three consecutive days of intraperitoneal administration of cyclophosphamide, mice in the CTX group displayed reduced activity and tendency to curl up, contrasting with the active and healthy state observed in other groups. By day 21, a noticeable decrease in body weight was evident in the CTX group compared to the Con group (*P* < 0.01). Interestingly, the middle surfactin group demonstrated higher body weight than both the other groups (*P* < 0.05) as well as the Con group (*P* < 0.05) (Fig. 1B). Moreover, the analysis of the immunological organ indices revealed a significant decrease in both thymus and spleen indices in the CTX group compared to the Con group (*P* < 0.01). Conversely, these indices exhibited a substantial increase in the middle surfactin group (*P* < 0.05) (Fig. 1C). Furthermore, the middle surfactin group demonstrated a considerable enhancement in phagocytic rate and index (monocyte-macrophage phagocytosis of chicken red blood cells) as well as splenic cell proliferation capacity when compared to the Con group (*P* < 0.05), with a highly significant difference relative to the CTX group (*P* < 0.01) (Fig. 1D and E). Notably, it should be emphasized that there was no linear relationship between surfactin dosage escalation and its immunoenhancing effect. Our finding strongly indicates that surfactin significantly enhances immune response in mice, with the middle surfactin group exhibiting particularly prominent immunoenhancing activity.

### Histological analysis of intestinal tissue in mice and evaluation of intestinal permeability

**Fig 2:**
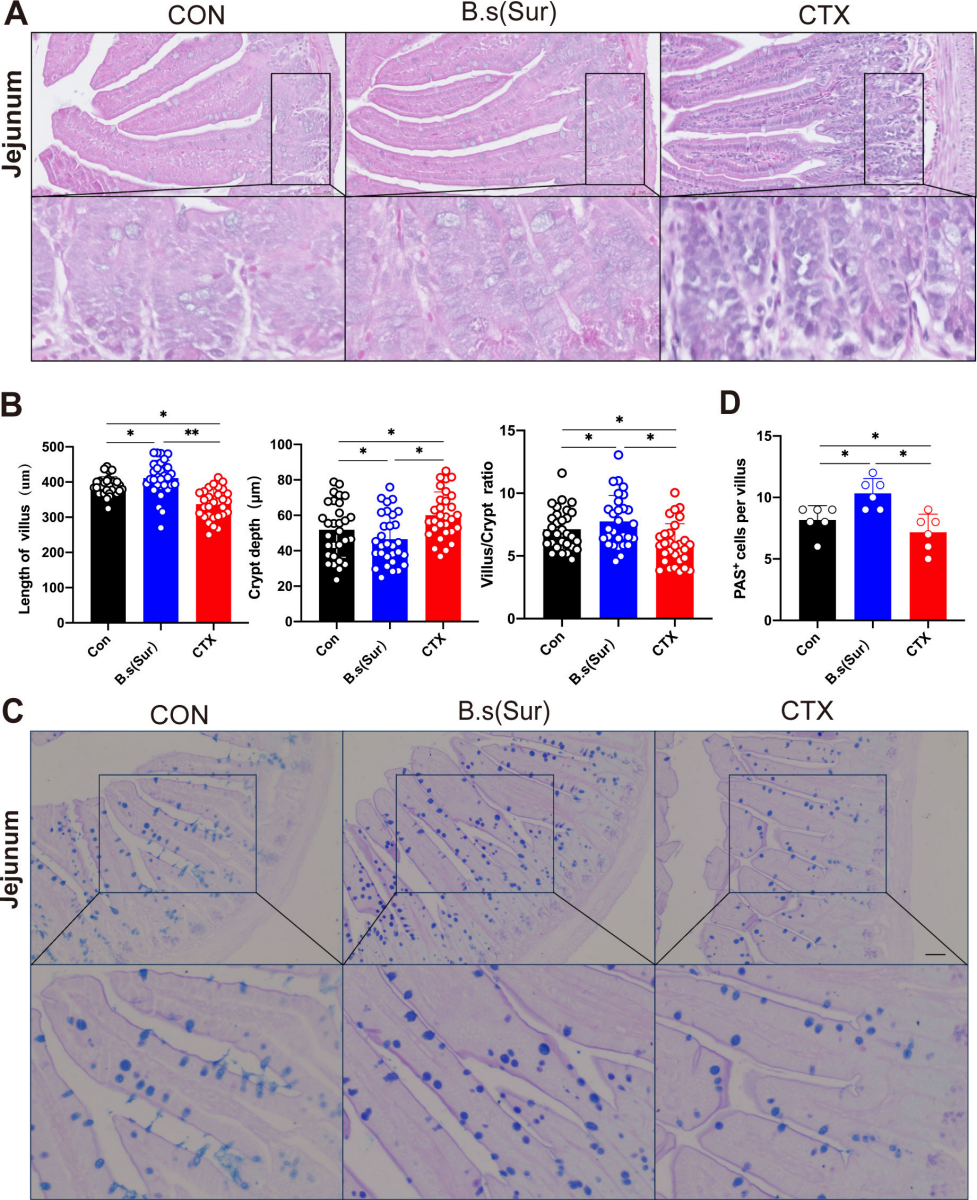
Histological analysis of intestinal tissue in mice and evaluation of intestinal permeability. (A) Representative pictures of H&E staining in the jejunum. Scale bar 50µm. (**B**)Graph showing jejunum villus height, crypt depth, and villus/crypt ratio. (**C**)Representative pictures of alcian blue staining in the Jejunum. Scale bar 100µm. (**D**)The graph shows jejunum tissue periodic acid-Schiff (PAS)+ cells per villus. Data represent the mean ± SD of two or three independent experiments; comparisons performed with *t*-tests (two groups). *P < 0.05, **P < 0.01. *n* = 6 per group.

To further investigate the immunomodulatory effects of the middle surfactin group on mice, we conducted a histological examination of the jejunum and colon architecture. As illustrated in Fig. 2, mice in the surfactin group exhibited well-organized and elongated jejunal villi, accompanied by a colon rich in glandular structures. In contrast, the CTX group displayed significant atrophy and disorganization of the jejunal villi. The colon exhibited disorganized mucosal layers, loosely arranged muscular layers, and damage to the serosal layer, accompanied by inflammatory infiltration (Fig. 2A; Fig. S1A). On the one hand, compared to the Con group, the CTX group demonstrated a substantial reduction in both jejunal villi height and villus-to-crypt (V/C) ratio (*P* < 0.01), as well as an increase in crypt depth (*P <* 0.05). On the other hand, when compared to the Con group, mice treated with intermediate doses of surfactin showed a notable increase in jejunal villi length (*P* < 0.05) and a decrease in crypt depth (*P* < 0.05), with a declining trend observed for V/C ratio (Fig. 2B). Moreover, histological examination of the CTX group’s colon using hematoxylin and eosin (H&E) staining revealed a significant presence of inflammatory infiltrates (Fig. S1B).

Additionally, goblet cell counts were assessed in the jejunum and colon sections through periodic acid-Schiff (PAS) and alcian blue staining. The results demonstrated a notable reduction in goblet cell numbers within the jejunal sections of the CTX group (*P* < 0.01). Meanwhile, the surfactin group exhibited a substantial increase in these cells compared to the Con group (Fig. 2C). All in all, our study indicated a significant downregulation of mucin-related gene *Muc2* and *TFF3* expression in the colon of the CTX group compared to the Con group (*P* < 0.01). In contrast, it was significantly upregulated in the surfactin group (Fig. S1C and D).

### Repercussions of surfactin on intestinal barrier-associated proteins in mice

**Fig 3:**
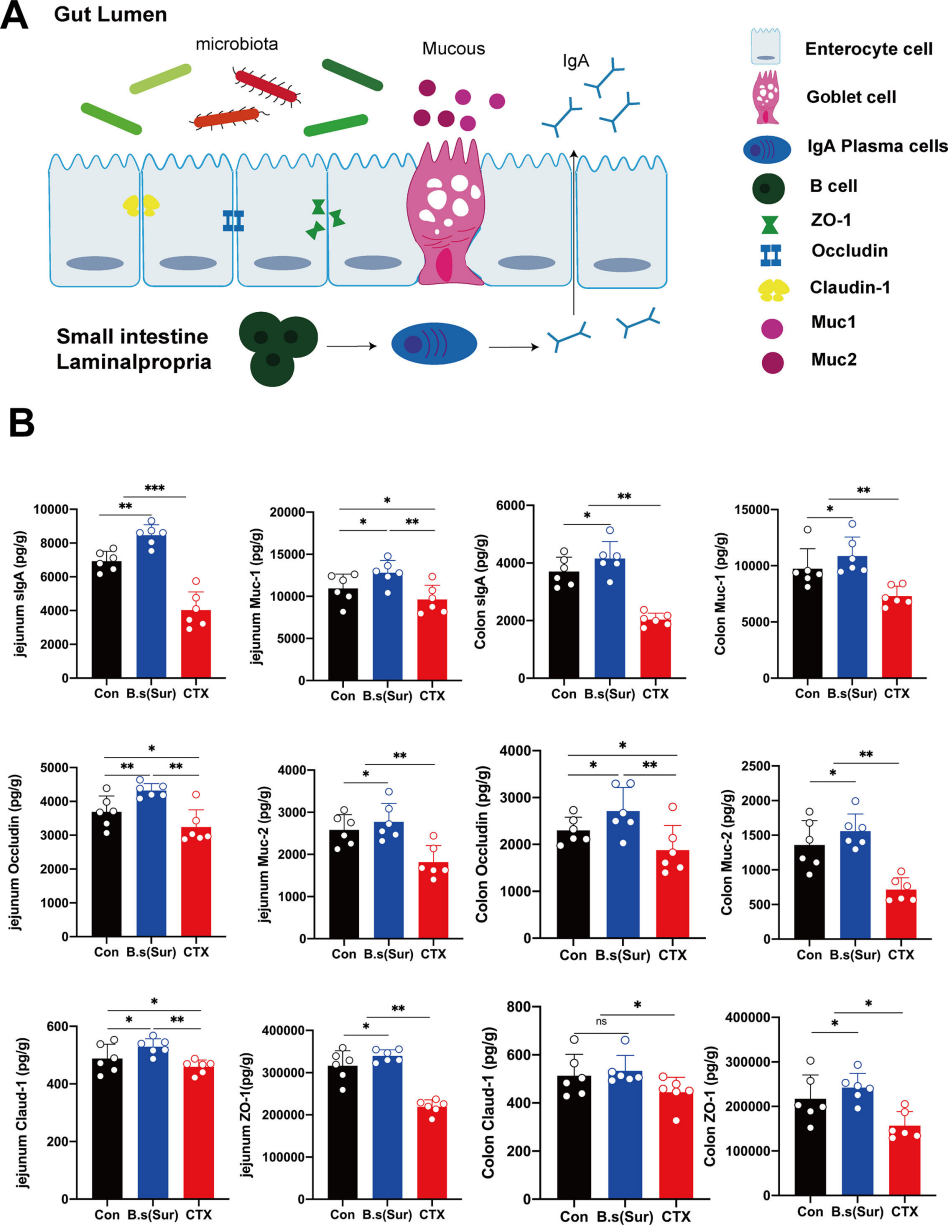
Repercussions of surfactin on intestinal barrier-associated proteins in mice. (A) Schematic representation of protein distribution in the lumen of the mouse intestine. (B）enzyme-linked immunosorbent assay of sIgA, Muc1, Muc2, occludin, claudin-1, and ZO-1 expression in the jejunum and colon. Data represent the mean ± SD of two or three independent experiments; comparisons performed with *t*-tests (two groups). *P < 0.05, **P < 0.01, ***P < 0.001,ns no statistical significance. *n* = 6 per group.

In this study, we investigated the effects of surfactin and CTX on the intestinal epithelium’s immune responses and barrier integrity in mice, focusing on key markers such as secretory IgA (sIgA), mucins, and tight junction proteins. The schematic illustrates the gut lumen and small intestine lamina propria, highlighting enterocyte cells, goblet cells, IgA plasma cells, and various tight junction proteins, including ZO-1, occludin, claudin-1, Muc1, and Muc2 (Fig. 3A). In the jejunum, surfactin treatment significantly increased the levels of secretory IgA, Muc1, and Muc2 proteins in the intestinal tract of mice, increasing by 1.7 times, 1.4 times, and 1.2 times compared to the control group, respectively (*P <* 0.05). This indicates that the mucosal immune function of the intestine was improved. Additionally, after surfactin treatment, the expression levels of the tight junction proteins occludin, claudin-1, and ZO-1 also significantly increased by 1.4 times, 1.3 times, and 1.4 times, respectively, compared to the control group, indicating enhanced intestinal barrier integrity. These proteins are crucial for maintaining tight junctions between intestinal cells, thereby preventing paracellular permeability.

In contrast, the levels of these indicators significantly decreased in the CTX-treated group compared to the control group: sIgA decreased by 2.1 times, Muc1 by 1.6 times, Muc2 by 1.4 times, occludin by 1.5 times, claudin-1 by 1.3 times, and ZO-1 by 1.5 times (*P <* 0.05), suggesting potential damage to the barrier integrity. A similar trend was observed in the colon. Compared to the control and CTX groups, surfactin treatment significantly increased the levels of sIgA, Muc1, and Muc2 (*P <* 0.05), accompanied by increased expression of occludin and ZO-1 (*P <* 0.05), further supporting the beneficial effects of surfactin on intestinal barrier function (Fig. 3B). These results indicate that surfactin not only enhances intestinal immune function but also maintains and strengthens the integrity of the intestinal barrier, thereby improving the defensive capability of the intestine.

In conclusion, surfactin significantly increased the expression of key immune- and barrier-associated proteins in the intestine, suggesting its potential role in improving intestinal immunity and barrier function. This provides scientific evidence for its application in promoting gastrointestinal health.

### Impact of intestinal microbial diversity on surfactin-treated mice

**Fig 4:**
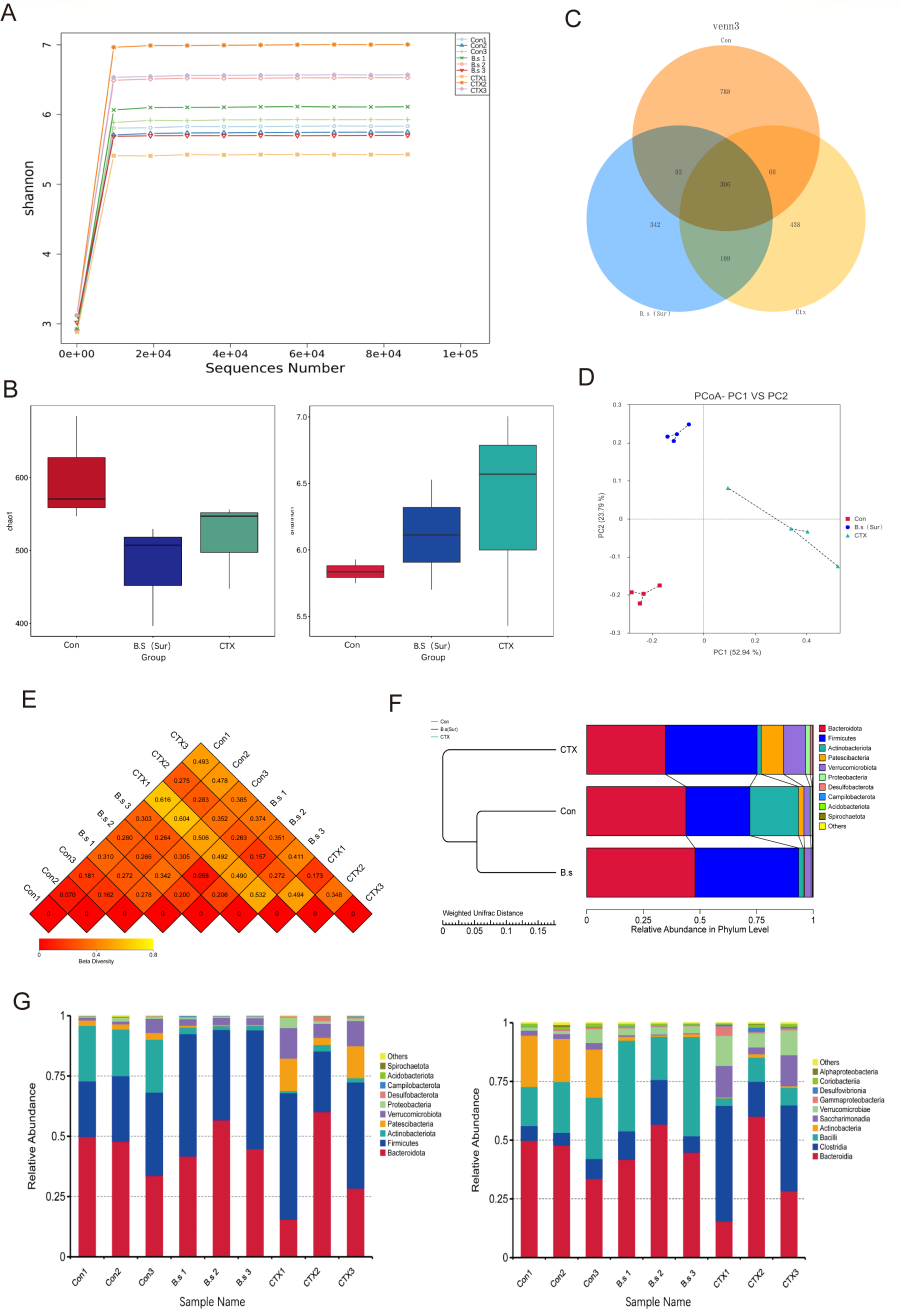
Impact of intestinal microbial diversity on surfactin P-treated mice. (A) Examples of species dilution curve. (**B**) Alphadiversity Chao and Shannon indexes. (**C**) Venndiagram showing the number of shared OTU and unique OTU in the three groups (**D**)Two-dimensional PCoA plots. (E) Beta diversity index unweighted unifrac distance matrix heatmap. (**F**)UPGMA clustering tree of weighted unifrac distances, integrating gate-level relative abundance of species across samples to display (**G**)average relative abundance of prevalent microbiota at the phylum and genus levels in different groups.*n* = 3 per group.

To explore the potential link between secretory protein expression and gut microbial community distribution, we conducted 16S rRNA sequencing analysis on fecal samples from mice to assess the impact of surfactin and CTX on the gut microbiome. First, alpha diversity analysis was performed to evaluate the abundance and richness of the gut microbiota. The Shannon diversity curve consistently indicated that our sequencing data captured a substantial portion of microbial diversity within the samples (Fig. 4A). Both the Chao1 and Shannon indices, which measure microbial community richness and diversity, respectively, showed no significant differences between experimental groups (Fig. 4B) (*P* > 0.05). However, the Venn diagram revealed distinct operational taxonomic units (OTUs) in the surfactin and CTX groups, identifying 342 and 438 unique OTUs, respectively (Fig. 4C). Second, beta diversity analysis revealed distinct differences between the CTX group and other groups, suggesting that CTX exposure resulted in significant alterations in the gut microbiota (Fig. 4D). Principal coordinate analysis (PCoA) and unweighted pair group method with arithmetic mean (UPGMA) further confirmed significant inter-group variations (Fig. S2A and B). Third, cluster analysis of the experimental samples showed that the surfactin group exhibited a clustering pattern more similar to the control group than the CTX group (Fig. 4E and F). At the phylum and genus levels, the composition and abundance of microbial communities were visualized using a sample clustering heatmap (Fig. 4G; Fig. S2C). Notably, the surfactin group exhibited an increased abundance of Firmicutes at the phylum level compared to the control group. This abundance is crucial for maintaining intestinal homeostasis by preserving the integrity of the intestinal barrier and supporting mucosal immunity. In contrast, the CTX group showed reduced Firmicutes abundance, potentially exacerbating susceptibility to mucosal immune dysfunction. At the genus level, there was a higher prevalence of *Lactobacillus* in the surfactin group compared to the control group, indicating an increase in beneficial bacteria associated with Firmicutes (Fig. S2E). Lastly, linear discriminant analysis of effect size (LEfSe) analysis integrated with LDA scores was employed to identify further significant disparities in bacterial abundance between groups (Fig. S2D), revealing that changes in the gut microbiota were closely associated with alterations in immune parameters across the different treatment regimens.

### The administration of surfactin ameliorated the immunosuppressive effects induced by cyclophosphamide in mice

**Fig 5:**
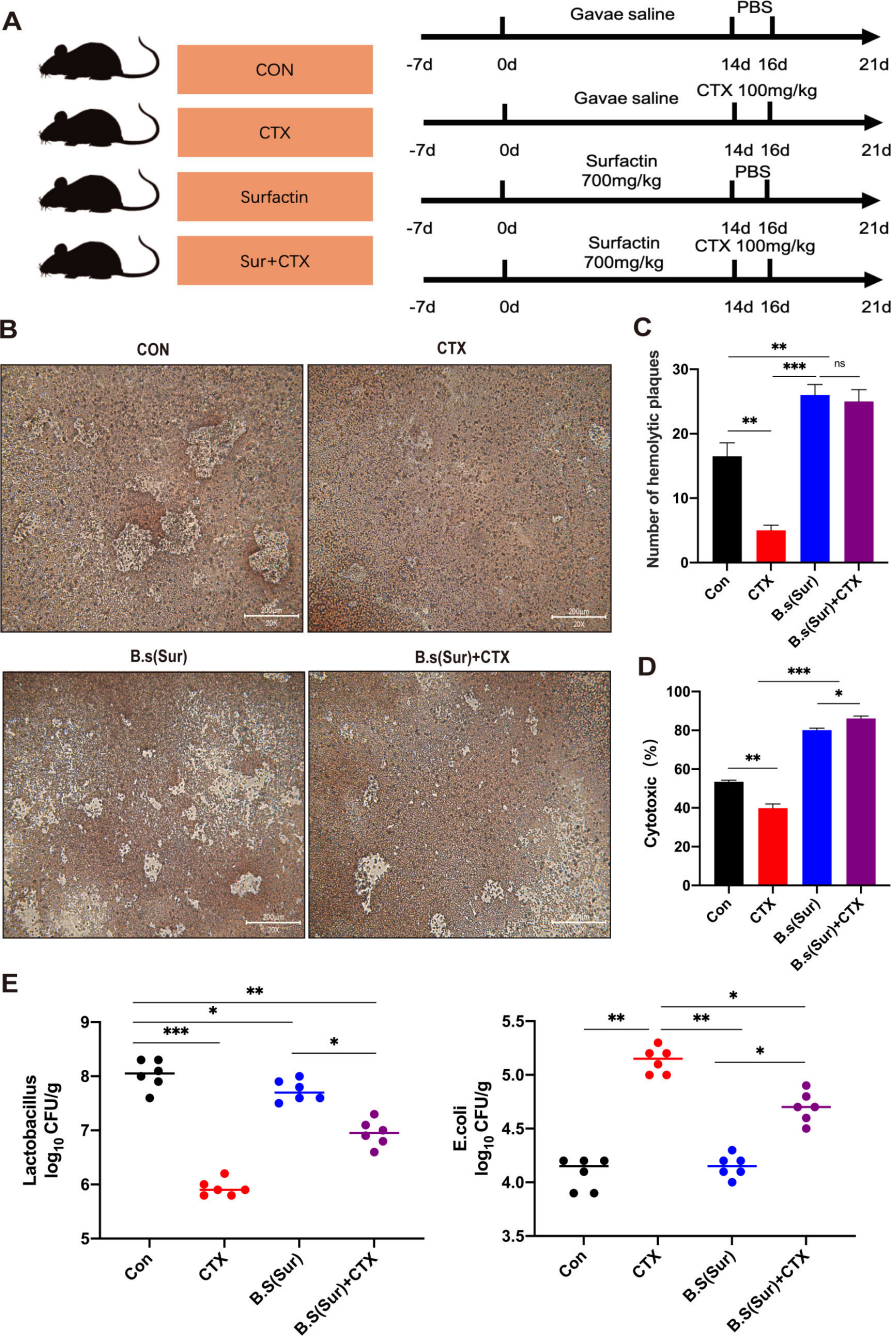
The administration of surfactin ameliorated the immunosuppressive effects induced by cyclophosphamide in mice. (A) Animal experiment design schematic. (**B**) Representative pictures of the antibody production and hemolytic plaque assay. (**C**) Graph showing antibody production and hemolytic plaque results. (**D**) Graph showing NK cytotoxicity results. (**E**)Number [log10(cfu/g)] of *Lactobacillus* in each group and number [log10(cfu/g)] of *E. coli* in each group. Data represent the mean ± SD of two or three independent experiments; comparisons performed with *t*-tests (two groups). *P < 0.05, **P < 0.01, ***P < 0.001,ns no statistical significance. *n* = 6 per group.

To investigate the impact of surfactin treatment on intestinal barrier function under CTX-induced conditions (Fig. 5A), cells isolated from all experimental groups of mice exhibited normal viability, with no significant differences observed (*P* > 0.05) (Fig. S3A and B). Hemolytic plaque assays revealed an increase in the number of hemolytic plaques in the surfactin treatment group compared to the CTX group, indicating a significant enhancement in overall antibody-secreting cell production within the spleen (Fig. 5B and C).

The cytotoxicity of NK cells, assessed by the lactate dehydrogenase (LDH) release assay, exhibited significantly enhanced NK cell activity in the surfactin treatment group compared to the CTX group (Fig. 5D). Periodic acid-Schiff and alcian blue staining demonstrated a decrease in goblet cells in the CTX group. In contrast, the surfactin treatment group displayed a higher abundance of goblet cells than other experimental groups, supported by elevated *Muc2* mRNA expression levels (Fig. S3C and D). Enzyme-linked immunosorbent assay (ELISA) analysis of colonic secretory protein levels confirmed that the surfactin treatment effectively ameliorated intestinal damage induced by CTX (*P <* 0.05) (Fig. S3E).

Furthermore, in comparison to the Con group, the CTX group exhibited a significant reduction in *Lactobacillus* counts (*P* < 0.05) and an elevation in *E. coli* counts within fecal samples (*P* < 0.05). However, the administration of surfactin treatment effectively reversed these alterations in gut microbiota composition induced by CTX, thereby restoring levels comparable to those observed in the Con group (Fig. 5E). The findings of this study suggest that pretreatment with surfactin contributes to the enhancement of intestinal mucosal barrier integrity, thereby alleviating the adverse effects of CTX-induced immunosuppression on gut health.

## DISCUSSION

Surfactin is extensively utilized in the livestock industry due to its environmentally friendly nature and biocompatibility. It can be incorporated into animal diets to improve weight gain in broiler chickens (11, 17), but precise dosage control is essential to prevent wastage or adverse effects. During the first 3 weeks of life, surfactin enhances productive performance by establishing a stable intestinal environment (18). Furthermore, it demonstrates effective antibacterial properties against pathogens such as *Clostridium perfringens* in growing pigs and finishers, offering an alternative to antibiotics (19). As an antimicrobial peptide, surfactin not only reduces the prevalence of pathogenic microbes, such as *Escherichia coli,* in the intestines but also promotes the presence of beneficial bacteria such as *lactobacilli*, thereby enhancing gut health in livestock and poultry (20–22). It is important to note that existing studies typically assess the beneficial effects of surfactin under normal physiological conditions or in response to pathogenic infections. However, our current understanding of the host-protective mechanisms of surfactin, particularly in immunosuppressed states, remains limited. Further comprehensive studies are needed to elucidate the immunomodulatory impacts of surfactin on the host.

CTX is a potent immunosuppressant commonly used for short-term stimulation to simulate models of immune suppression and evaluate intestinal health (6). Our study has found that surfactin improves the morphological structure of the intestine and enhances nutrient absorption in mice. The indices of immune organs in mice showed significant increases; however, these increments did not show a linear correlation with the dosage of surfactin, suggesting a positive impact on mouse health without implying absolute superiority in dietary inclusion. Research has demonstrated that in acute oral toxicity tests on rats, no mortalities or adverse behavioral changes were observed even at high 2,000 mg/kg of surfactin doses. Additionally, no abnormalities or signs of toxicity were noted in the animals’ behavior. In a 28-day subacute toxicity study, the oral administration of 500 mg/kg of surfactin did not result in any toxic symptoms, pathological changes, or abnormal blood biochemical indicators (23). Moreover, in bone marrow micronucleus tests, surfactin at oral doses of up to 4,000 mg/kg showed no genotoxic effects in male ICR mice. When pregnant female mice were given 500 mg/kg of surfactin for 12 consecutive days, there were no changes in the relative mass of maternal organs (such as the brain, spleen, kidneys, liver, and heart) nor were there any signs of fetal toxicity or teratogenic effects (24). Furthermore, the long-term oral administration of 10 mg/kg of surfactin in ICR mice demonstrated high biosafety, with potential applications as an emulsifier in food production and processing (25). These studies collectively underscore the overall safety of surfactin at the doses tested. Our findings align with these reports, reinforcing surfactin’s potential as a safe and effective compound for various applications. However, given that this study utilized C57BL6/J mice, further research is required to determine the long-term safety of surfactin in this and other models. Furthermore, macrophages play a crucial role in non-specific immunity and bridge innate and adaptive immunity (26–28). Surfactin was observed to enhance the phagocytic ability of peritoneal macrophages in mice and promote the proliferation of T and B lymphocytes, thereby improving cellular and humoral immune capacities.

The gut’s primary defense mechanism, an intestinal barrier, primarily composed of mucins secreted by goblet cells (29, 30), is the gut’s primary defense mechanism. Muc2 is predominantly released by goblet cells, while Muc1 is synthesized by epithelial cells. AB-PAS staining revealed a significant decrease in the number of goblet cells on the villi of the jejunum in the CTX group compared to the Con group. In contrast, the surfactin-treated group exhibited a notable increase. These findings were consistent with qPCR and ELISA results obtained from colon samples.

Furthermore, sIgA, mainly produced by plasma cells beneath the intestinal mucosa, forms a protective layer over the intestinal epithelium to prevent direct contact between pathogens, exogenous antigens, and epithelial cells. It also interacts with beneficial bacteria to enhance their survival while inhibiting harmful bacterial growth (31, 32). Surfactin treatment significantly elevated sIgA secretion levels in mice’s jejunum and colon, thereby providing protective effects. Studies indicate that CTX can impact intestinal permeability by inhibiting the synthesis of tight junction proteins (16). Occludin and claudin-1 are transmembrane proteins that play pivotal roles in cell adhesion and keratinocyte proliferation. ZO-1 is a peripheral membrane protein indispensable for tight junction assembly (33–35). Surfactin enhances the expression of tight junction proteins in both the jejunum and colon of mice, thereby reducing intestinal permeability and positively contributing to maintaining intestinal barrier integrity.

The gut microbiota plays a pivotal role in maintaining host immune homeostasis, and its disruption can lead to significant health issues, including inflammation and compromised gut barrier function (36, 37). In our study, cyclophosphamide administration resulted in a notable shift in gut microbiota composition, characterized by an increased abundance of *Escherichia coli*, often associated with intestinal inflammation (38). This aligns with previous findings that highlight the negative impact of CTX on gut microbial balance and its subsequent effects on gut health (39). Alpha diversity analysis using 16S rRNA sequencing revealed no significant differences between the experimental groups regarding overall microbial diversity, as evidenced by the Shannon and Chao1 indices. However, beta diversity analysis and cluster analysis demonstrated that the gut microbiota of the surfactin-treated group closely resembled that of the control group, indicating that surfactin helped maintain a more stable gut microbiome composition compared to the CTX-treated group.

Further examination of the microbiota at the phylum and genus levels revealed that surfactin treatment was associated with an increased prevalence of beneficial phyla such as Firmicutes and Bacteroidetes. These phyla include specific genera, such as *Lactobacillus*, *Ruminococcus*, and *Enterococcus*, known for their roles in producing short-chain fatty acids, promoting intestinal angiogenesis, and aiding digestion, respectively. These beneficial bacteria are crucial for maintaining gut homeostasis and supporting intestinal barrier integrity (40–42).

In contrast, the CTX group exhibited a reduction in these beneficial taxa, which likely contributed to the observed disruptions in gut homeostasis and increased susceptibility to inflammation. The LEfSe and LDA analyses further validated the distinct microbial profiles among the experimental groups, with the surfactin group showing a microbial composition more aligned with health-associated features. Our results highlight surfactin’s critical role in maintaining gut microbiome stability, particularly against CTX-induced disruptions, and its potential as a therapeutic regulator of gut health. Currently, there is limited research on the role of surfactin in mitigating immunosuppression-induced damage in mice. Our study revealed that the group treated with surfactin exhibited a reversal of the decline in antibody production caused by the CTX group, highlighting the intricate and precise nature of the adaptive immune system. Additionally, surfactin treatment significantly restored NK cell activity in mice. NK cells are an integral part of the innate immune system and function independently of antigen specificity by recognizing target cells through various surface receptors. These receptors play a crucial role in detecting changes in cell surface molecules (43, 44), essential for maintaining overall health and defending against diseases. Furthermore, the administration of surfactin reduced *Escherichia coli* levels while increasing lactobacilli and other beneficial digestive bacteria populations in mouse feces. This indicates that surfactin is regulated in modulating gut microbiota composition and maintaining intestinal homeostasis.

**Fig 6:**
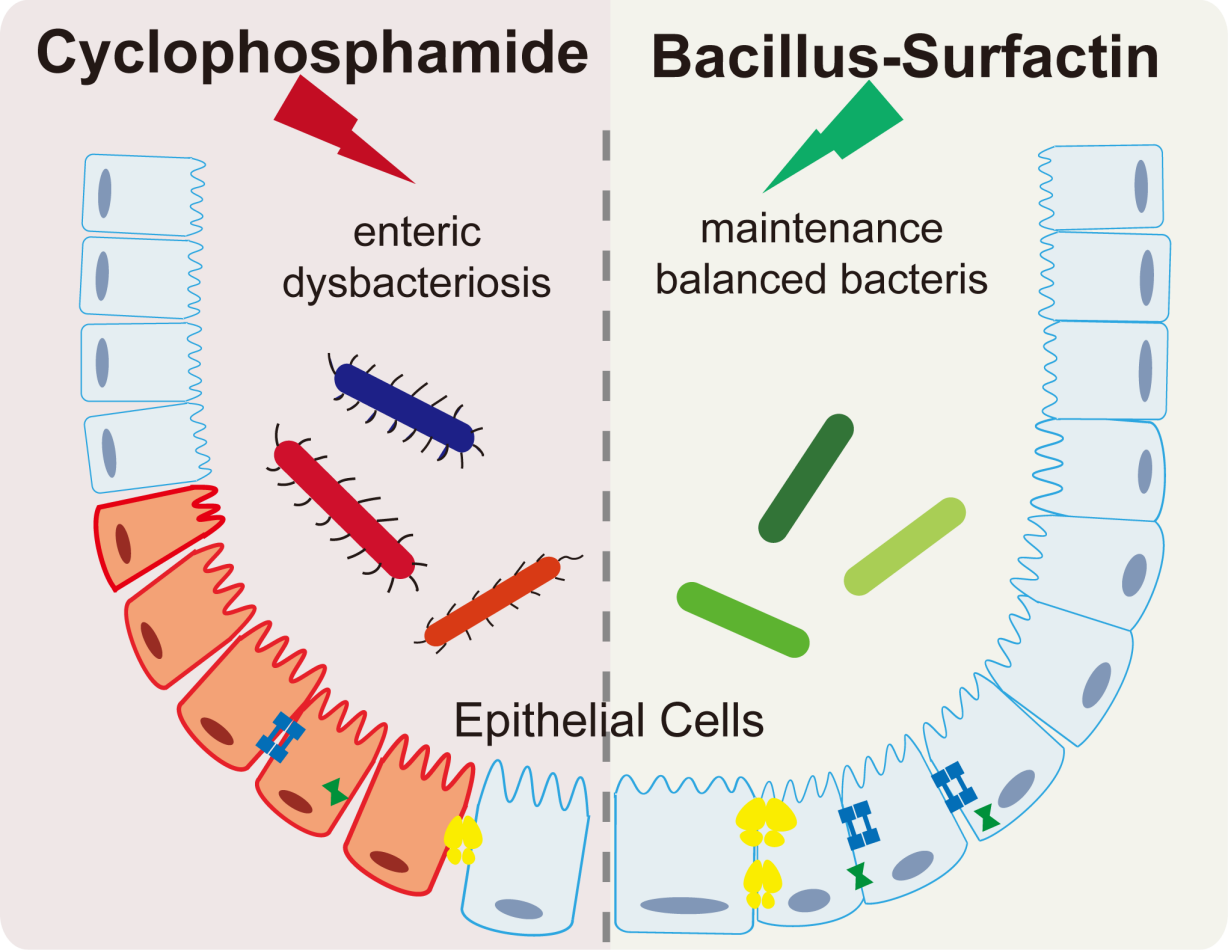
Surfactin and its relationship with gut microbiota. The impacts of surfactin on the gut microbiome are delineated, encompassing modulation of microbial composition, influence on bacterial colonization, and regulation of intestinal immunity.

In summary, this study demonstrates that surfactin enhances immune cell functionality as an immunomodulator, regulates gut microbiome composition (Fig. 6), and can prevent weight loss and intestinal inflammation induced by CTX-mediated immunosuppression.

## MATERIALS AND METHODS

### Animals

C57BL/6J mice were procured from the Animal Research Center of Yangzhou University and maintained under specific pathogen-free conditions at 25°C with a standard 12/12-hour light/dark cycle, provided *ad libitum* access to food and water, and acclimatized to our facility for 1 week. Male mice aged between 6 and 8 weeks were used in the study, with the exact number specified in the figure legends. All animal experiments were conducted following approval by the Institutional Animal Care and Use Committee of Nanjing Agricultural University (NJAU.No20221215244). Each of the animals was humanely euthanized by cervical dislocation, and the spleen and thymus of mice were weighed and used in the following experiments.

### Experimental design and treatment protocol

Experiment I (Fig. 1 to 4), Immunoimmunoassay of Mice With Oral Surfactin, experimental design is presented in Fig. 1A. Following the adaptation period, the 75 mice were randomly divided into one of the following groups: blank group (administered orally with distilled water daily) and CTX group (administered orally with distilled water daily, intraperitoneally with 100mg/kg cyclophosphamide on days 14–16). The experimental groups received low (orally administered at 350mg/kg daily for 14 days), middle (orally administered at 700mg/kg daily for 14 days), and high (orally administered at 2,100mg/kg daily for 14 days). Additionally, a treatment group was given 700mg/kg, administered for 14 days, and injected with 100mg/kg cyclophosphamide on days 14–16. Throughout the treatment period, changes in body weight were monitored daily. At the end of this period, cervical dislocation was used to euthanize the mice. Mice were randomly selected for each group, and the small intestine, large intestine, thymus, and spleen were removed and weighed accordingly.

Experiment II (Fig. 5), Orally Surfactin Treatment in Mice for CTX-Induced Immunosuppression. The 24 mice were randomly divided into 4 groups. The experimental design is shown in Fig. 5A, and three were randomly selected from each group for the experiment. Beijing Enhalor International Tech Co., Ltd provided the *B.s* surfactin utilized in this experiment.

### Isolation and analysis of peritoneal macrophages

Mice were intraperitoneally injected with 2 mL of 1% chicken erythrocyte suspension, and the abdomen was gently massaged to disperse the cells. After 25–30 minutes, mice were euthanized by cervical dislocation. Then, 2 mL of physiological saline was injected into the peritoneal cavity. A drop of physiological saline was placed on a clean glass slide, to which a drop of peritoneal fluid was added. The slide was left undisturbed for 10 minutes to allow peritoneal macrophages to adhere to the slide. The saline was discarded, and the slide was left to dry slightly before being stained with Wright’s stain. For Wright’s staining, three to four drops of the paint were applied to the specimen, enough to cover it completely. After 5 minutes, the excess stain was shaken off the slide, which was then gently washed with tap water and blotted dry with absorbent paper. The dried slide was examined under oil immersion.

### Proliferation assay of splenic lymphocytes

The mouse’s spleen was ground through a 70-µm sieve, and 4–5 mL of the splenic cell suspension was immediately transferred to a 15-mL centrifuge tube. The sample was centrifuged at 800 g for 30 minutes, removed the lymphocyte layer, and lysed red blood cells. Then, 10 mL of RPMI 1640 medium was added to wash the cells. After centrifugation at 250 g for 10 minutes, the cells were collected. The supernatant was discarded, and the splenic lymphocytes were resuspended. The trypan blue exclusion method determined cell viability (≥95%).

Splenocytes were seeded at a density of 5 × 10^5^ cells per well in a 96-well flat-bottomed microplate. Concanavalin A (ConA, Sigma c5275) was added to each well at a concentration of 2.5 mg per well. Cells were incubated in RPMI 1640 medium without serum as control with a total volume of 200 mL per well. After incubation for 24 hours, cell proliferation was evaluated using the CCk8 assay.

### LDH release assay for NK cell activity measurement

NK cells were isolated from mouse spleen cells at a density of 1 × 10^6^ cells per well, and YAC-1 cells were cultured at a density of 1 × 10^7^ cells per well. The Lactate Dehydrogenase Activity Assay Kit (Elabscience E-BC-K046-M) was utilized to monitor LDH activity, and the absorbance (A) of each well was measured at a wavelength of 450 nm using an enzyme-linked immunosorbent assay reader.

### Revising the histological analysis of mouse intestines

The mice were euthanized on the final day of the animal experiment following a fasting period. Samples required for the experiments were collected, and a portion of these samples (specifically, jejunum and distal colon) was immersed in 4% paraformaldehyde overnight and subsequently embedded in paraffin. Tissue sections were obtained and stained with H&E or alcian blue-PAS. The tissue morphology was observed under a light microscope at magnifications of 40× or 100×. Image J software quantified goblet cells and their corresponding colonic mucosa area. Additionally, other samples were stored at −70°C for future investigations. Cytokine levels in jejunal and colonic tissue homogenates were measured using ELISA kits specific to mouse sIgA, Muc-1, Muc-2, occludin, claudin-1, and ZO-1 (Jiangsu Meimian Industrial Co., Ltd.), following the manufacturer’s instructions.

### Extraction of DNA from mouse feces and subsequent analysis of the 16S rRNA gene sequencing

The fecal samples were cryopreserved in dry ice and shipped to Beijing Novozymes Bioinformatics Co. for 16S rRNA amplification targeting the V3-V4 region. The amplification of the V3-V4 variable region of enteric bacterial 16S DNA was performed using universal primers, forward 338F (5′-ACTCCTACGGGGAGGCAGCAG-3′) and reverse 806R (5′-GGACTACHVGGGTWTCTAAT-3′), as per the manufacturer’s instructions. Each sample was labeled with a unique barcode using polymerase chain reaction. The PCR products were then separated on a 2% agarose gel and quantified using a NanoDrop ND-2000 spectrophotometer (Thermo Scientific Inc., USA). PCR products of the same mass from each sample were pooled together based on their relative concentrations to construct the library. Sequencing was performed on the Illumina MiSeq platform (San Diego, CA, USA) following the manufacturer’s instructions to generate 250-base paired-end reads. Raw paired-end reads were processed using the QIIME 2 platform (version 2020.2). Sequence quality control was conducted using DADA2: raw reads underwent filtering, trimming, denoising, and deduplication; forward and reverse sequences were merged; and chimeras were eliminated. Alpha and beta diversity analyses were also carried out using QIIME 2. Alpha diversity measures and statistics were calculated utilizing the Shannon index. Beta diversity was assessed by employing the Bray-Curtis dissimilarity metric. Linear discriminant analysis of effect size was employed to identify significantly different genera between groups, with data analysis being performed on the Novozymes cloud platform, including species composition, species differences, and environmental correlation analysis.

### The process of RNA isolation and subsequent gene expression analysis

Total RNA was extracted from intestinal tissues using the RNAiso Plus Kit (Taraka, China). Subsequently, cDNA synthesis was performed on the extracted RNA using reverse transcription reagents (Takara, China). The resulting cDNA templates were amplified using appropriate primers on the QuantStudio 6 Flex system (Applied Biosystems, USA). The GAPDH gene served as an internal reference with primer sequences listed in Table 1. This study analyzed mRNA levels using the 2^−ΔΔCT^ method.

**TABLE 1:** Sequence of primers used in this study

Gene	Sequence (5′−3′）	Accession no.
GAPDH	F: ATGGTGAAGGTCGGTGTGAA R: TGGAAGATGGTGATGGGCTT	XM_017321385.2
*Tff3*	F: ATTACGTTGGCCTGTCTCCA R: CGATGTGACAGAGGGGTAGC	NM_011575.2
*Muc2*	F: TCCAGGTCTCGACATTAGCAG R: GTGCTGAGAGTTTGCGTGTCT	NM_001145874.1

### Statistical analysis

In all experiments, the data were expressed as mean ± SEM. GraphPad Prism 5.0 software was utilized to plot and compare multiple groups using one-way analysis of variance. *P* < 0.05 indicates that the difference is statistically significant: *P < 0.05, **P < 0.01, ***P < 0.001, and ns (no statistical significance, *P* > 0.05).

## Data Availability

The 16S sequences obtained in this study have been deposited in NCBI, and the accession number PRJNA1054753 has been assigned.

## SUPPLEMENTAL MATERIAL

Supplemental figures spectrum.00918-24-s0001.docx Figures S1 to S3.

ASM does not own the copyrights to Supplemental Material that may be linked to, or accessed through, an article. The authors have granted ASM a non-exclusive, world-wide license to publish the Supplemental Material files. Please contact the corresponding author directly for reuse.

## References

[1] Liu JP, Wang J, Zhou SX, Huang DC, Qi GH, Chen GT. 2022. Ginger polysaccharides enhance intestinal immunity by modulating gut microbiota in cyclophosphamide-induced immunosuppressed mice. Int J Biol Macromol 223:1308–1319. doi:10.1016/j.ijbiomac.2022.11.104 36395935

[2] Bao X, Wu J. 2021. Impact of food-derived bioactive peptides on gut function and health. Food Res Int 147:110485. doi:10.1016/j.foodres.2021.110485 34399481

[3] Ying M, Yu Q, Zheng B, Wang H, Wang J, Chen S, Nie S, Xie M. 2020. Cultured Cordyceps sinensis polysaccharides modulate intestinal mucosal immunity and gut microbiota in cyclophosphamide-treated mice. Carbo Polym 235:115957. doi:10.1016/j.carbpol.2020.115957 32122493

[4] Chen S, Wang J, Fang Q, Dong N, Fang Q, Cui SW, Nie S. 2021. A polysaccharide from natural Cordyceps sinensis regulates the intestinal immunity and gut microbiota in mice with cyclophosphamide-induced intestinal injury. Food Funct 12:6271–6282. doi:10.1039/d1fo00596k 34105571

[5] Zeng Z, Huang Z, Yue W, Nawaz S, Chen X, Liu J. 2023. Lactobacillus plantarum modulate gut microbiota and intestinal immunity in cyclophosphamide-treated mice model. Biomed Pharmacother 169:115812. doi:10.1016/j.biopha.2023.115812 37979376

[6] Ahlmann M, Hempel G. 2016. The effect of cyclophosphamide on the immune system: implications for clinical cancer therapy. Cancer Chemother Pharmacol 78:661–671. doi:10.1007/s00280-016-3152-1 27646791

[7] Yang J, Liu K, Qu J, Wang X. 2013. The changes induced by cyclophosphamide in intestinal barrier and microflora in mice. Eur J Pharmacol 714:120–124. doi:10.1016/j.ejphar.2013.06.006 23791611

[8] Song X, Liu L, Peng S, Liu T, Chen Y, Jia R, Zou Y, Li L, Zhao X, Liang X, Tang H, Yin Z. 2022. Resveratrol regulates intestinal barrier function in cyclophosphamide‐induced immunosuppressed mice. J Sci Food Agric 102:1205–1215. doi:10.1002/jsfa.11458 34346509

[9] Tian B, Wang P, Xu T, Cai M, Mao R, Huang L, Sun P, Yang K. 2023. Ameliorating effects of Hericium erinaceus polysaccharides on intestinal barrier injury in immunocompromised mice induced by cyclophosphamide. Food Funct 14:2921–2932. doi:10.1039/d2fo03769f 36892225

[10] Sumi CD, Yang BW, Yeo IC, Hahm YT. 2015. Antimicrobial peptides of the genus Bacillus: a new era for antibiotics. Can J Microbiol 61:93–103. doi:10.1139/cjm-2014-0613 25629960

[11] Liu J, Wang X, Shi W, Qian Z, Wang Y. 2019. Sensitization of avian pathogenic Escherichia coli to amoxicillin in vitro and in vivo in the presence of surfactin. PLoS One 14:e0222413. doi:10.1371/journal.pone.0222413 31513649 PMC6742356

[12] Yuan L, Zhang S, Peng J, Li Y, Yang Q. 2019. Synthetic surfactin analogues have improved anti-PEDV properties. PLoS One 14:e0215227. doi:10.1371/journal.pone.0215227 30973929 PMC6459484

[13] Chen X, Zhao H, Lu Y, Meng F, Lu Z, Lu Y. 2023. Surfactin mitigates dextran sodium sulfate-induced colitis and behavioral disorders in mice by mediating gut–brain-axis balance. J Agric Food Chem 71:1577–1592. doi:10.1021/acs.jafc.2c07369 36634244

[14] de Jonge ME, Huitema ADR, Rodenhuis S, Beijnen JH. 2005. Clinical pharmacokinetics of cyclophosphamide. Clin Pharmacokinet 44:1135–1164. doi:10.2165/00003088-200544110-00003 16231966

[15] Cui Y, Zhang L, Lu C, Dou M, Jiao Y, Bao Y, Shi W. 2022. Effects of compound small peptides of Chinese medicine on intestinal immunity and cecal intestinal flora in CTX immunosuppressed mice. Front Microbiol 13:959726. doi:10.3389/fmicb.2022.959726 35958151 PMC9358959

[16] Cui Y, Zhang L, Liu Y, Liu W, Shi W, Bao Y. 2023. Compound small peptide of Chinese medicine alleviates cyclophosphamide induced immunosuppression in mice by Th17/Treg and jejunum intestinal flora. Front Microbiol 14:1039287. doi:10.3389/fmicb.2023.1039287 37056742 PMC10089124

[17] Kowall M, Vater J, Kluge B, Stein T, Franke P, Ziessow D. 1998. Separation and characterization of Surfactin isoforms produced by Bacillus subtilis OKB 105. J Colloid Interface Sci 204:1–8. doi:10.1006/jcis.1998.5558 9665760

[18] Horng Y-B, Yu Y-H, Dybus A, Hsiao FS-H, Cheng Y-H. 2019. Antibacterial activity of Bacillus species-derived surfactin on Brachyspira hyodysenteriae and Clostridium perfringens. AMB Expr 9:188. doi:10.1186/s13568-019-0914-2 PMC6872690 31754906

[19] Plata G, Baxter NT, Susanti D, Volland-Munson A, Gangaiah D, Nagireddy A, Mane SP, Balakuntla J, Hawkins TB, Kumar Mahajan A. 2022. Growth promotion and antibiotic induced metabolic shifts in the chicken gut microbiome. Commun Biol 5:293. doi:10.1038/s42003-022-03239-6 35365748 PMC8975857

[20] Cheng YH, Zhang N, Han JC, Chang CW, Hsiao FS, Yu YH. 2018. Optimization of surfactin production from Bacillus subtilis in fermentation and its effects on Clostridium perfringens ‐induced necrotic enteritis and growth performance in broilers. Anim Physiol Nutr 102:1232–1244. doi:10.1111/jpn.12937 29901824

[21] Cheng YH, Horng YB, Dybus A, Yu YH. 2021. Bacillus licheniformis-fermented products improve growth performance and intestinal gut morphology in broilers under Clostridium perfringens challenge. J Poult Sci 58:30–39. doi:10.2141/jpsa.0200010 33519284 PMC7837812

[22] Chen YC, Yu YH. 2020. Bacillus licheniformis–fermented products improve growth performance and the fecal microbiota community in broilers. Poult Sci 99:1432–1443. doi:10.1016/j.psj.2019.10.061 32115030 PMC7587626

[23] Hwang Y-H, Kim M-S, Song I-B, Park B-K, Lim J-H, Park S-C, Yun H-I. 2009. Subacute (28 day) toxicity of Surfactin C, a lipopeptide produced by Bacillus subtilis, in rats. J HEALTH Sci (El Monte) 55:351–355. doi:10.1248/jhs.55.351

[24] Hwang Y-H, Park B-K, Lim J-H, Kim M-S, Song I-B, Park S-C, Yun H-I. 2008. Evaluation of genetic and developmental toxicity of Surfactin C from Bacillus subtilis BC1212. J HEALTH Sci (El Monte) 54:101–106. doi:10.1248/jhs.54.101

[25] Santos VSV, Silveira E, Pereira BB. 2018. Toxicity and applications of surfactin for health and environmental biotechnology. J Toxicol Environ Health B Crit Rev 21:382–399. doi:10.1080/10937404.2018.1564712 30614421

[26] Na YR, Stakenborg M, Seok SH, Matteoli G. 2019. Macrophages in intestinal inflammation and resolution: a potential therapeutic target in IBD. Nat Rev Gastroenterol Hepatol 16:531–543. doi:10.1038/s41575-019-0172-4 31312042

[27] Meli VS, Veerasubramanian PK, Atcha H, Reitz Z, Downing TL, Liu WF. 2019. Biophysical regulation of macrophages in health and disease. J Leukoc Biol 106:283–299. doi:10.1002/JLB.MR0318-126R 30861205 PMC7001617

[28] Park MD, Silvin A, Ginhoux F, Merad M. 2022. Macrophages in health and disease. Cell 185:4259–4279. doi:10.1016/j.cell.2022.10.007 36368305 PMC9908006

[29] Paone P, Cani PD. 2020. Mucus barrier, mucins and gut microbiota: the expected slimy partners? Gut 69:2232–2243. doi:10.1136/gutjnl-2020-322260 32917747 PMC7677487

[30] Jakobsson HE, Rodríguez-Piñeiro AM, Schütte A, Ermund A, Boysen P, Bemark M, Sommer F, Bäckhed F, Hansson GC, Johansson MEV. 2015. The composition of the gut microbiota shapes the colon mucus barrier. EMBO Rep 16:164–177. doi:10.15252/embr.201439263 25525071 PMC4328744

[31] Pabst O, Izcue A. 2022. Secretory IgA: controlling the gut microbiota. Nat Rev Gastroenterol Hepatol 19:149–150. doi:10.1038/s41575-021-00563-w 34862512

[32] Schofield WB, Palm NW. 2018. Gut microbiota: IgA protects the pioneers. Curr Biol 28:R1117–R1119. doi:10.1016/j.cub.2018.08.019 30253156

[33] Steed E, Balda MS, Matter K. 2010. Dynamics and functions of tight junctions. Trends Cell Biol 20:142–149. doi:10.1016/j.tcb.2009.12.002 20061152

[34] Balda MS, Whitney JA, Flores C, González S, Cereijido M, Matter K. 1996. Functional dissociation of paracellular permeability and transepithelial electrical resistance and disruption of the apical-basolateral intramembrane diffusion barrier by expression of a mutant tight junction membrane protein. J Cell Biol 134:1031–1049. doi:10.1083/jcb.134.4.1031 8769425 PMC2120963

[35] Balda MS, Matter K. 2000. The tight junction protein ZO-1 and an interacting transcription factor regulate ErbB-2 expression. EMBO J 19:2024–2033. doi:10.1093/emboj/19.9.2024 10790369 PMC305688

[36] Brenchley JM, Douek DC. 2012. Microbial translocation across the GI tract. Annu Rev Immunol 30:149–173. doi:10.1146/annurev-immunol-020711-075001 22224779 PMC3513328

[37] Ishikawa D, Sasaki T, Osada T, Kuwahara-Arai K, Haga K, Shibuya T, Hiramatsu K, Watanabe S. 2017. Changes in intestinal microbiota following combination therapy with fecal microbial transplantation and antibiotics for ulcerative colitis. Inflamm Bowel Dis 23:116–125. doi:10.1097/MIB.0000000000000975 27893543

[38] Xu X, Zhang X. 2015. Effects of cyclophosphamide on immune system and gut microbiota in mice. Microbiol Res 171:97–106. doi:10.1016/j.micres.2014.11.002 25553830

[39] Liu Y, Wu Y, Wu J, Li X, Yu L, Xie K, Zhang M, Ren L, Ji Y, Li Y. 2022. Exposure to veterinary antibiotics via food chain disrupts gut microbiota and drives increased Escherichia coli virulence and drug resistance in young adults. Pathogens 11:1062. doi:10.3390/pathogens11091062 36145494 PMC9500718

[40] Riazi-Rad F, Behrouzi A, Mazaheri H, Katebi A, Ajdary S. 2021. Impact of gut microbiota on immune system. Acta Microbiol Immunol Hung 68:135–144. doi:10.1556/030.2021.01532 34375301

[41] Han Y, Zhang Y, Ouyang K, Chen L, Zhao M, Wang W. 2022. Sulfated Cyclocarya paliurus polysaccharides improve immune function of immunosuppressed mice by modulating intestinal microbiota. Int J Biol Macromol 212:31–42. doi:10.1016/j.ijbiomac.2022.05.110 35597376

[42] Crost EH, Coletto E, Bell A, Juge N. 2023. Ruminococcus gnavus: friend or foe for human health. FEMS Microbiol Rev 47:fuad014. doi:10.1093/femsre/fuad014 37015876 PMC10112845

[43] Rückert T, Lareau CA, Mashreghi M-F, Ludwig LS, Romagnani C. 2022. Clonal expansion and epigenetic inheritance of long-lasting NK cell memory. Nat Immunol 23:1551–1563. doi:10.1038/s41590-022-01327-7 36289449 PMC9663309

[44] Rascle P, Woolley G, Jost S, Manickam C, Reeves RK. 2023. NK cell education: physiological and pathological influences. Front Immunol 14:1087155. doi:10.3389/fimmu.2023.1087155 36742337 PMC9896005

