# Supplementary material for: Surfactin from Bacillus subtilis enhances immune response and contributes to the maintenance of intestinal microbial homeostasis
Source: Microbiol Spectr. 2024 Oct 29;12(12):e00918-24. doi: 10.1128/spectrum.00918-24 (PMC11619528; doi:10.1128/spectrum.00918-24)
Supplement: Supplemental figures — Figures S1 to S3. [file spectrum.00918-24-s0001.docx]

**Supplementary Information**

**Figure S1: Histological analysis of colon section in mice and evaluation of intestinal permeability**

**
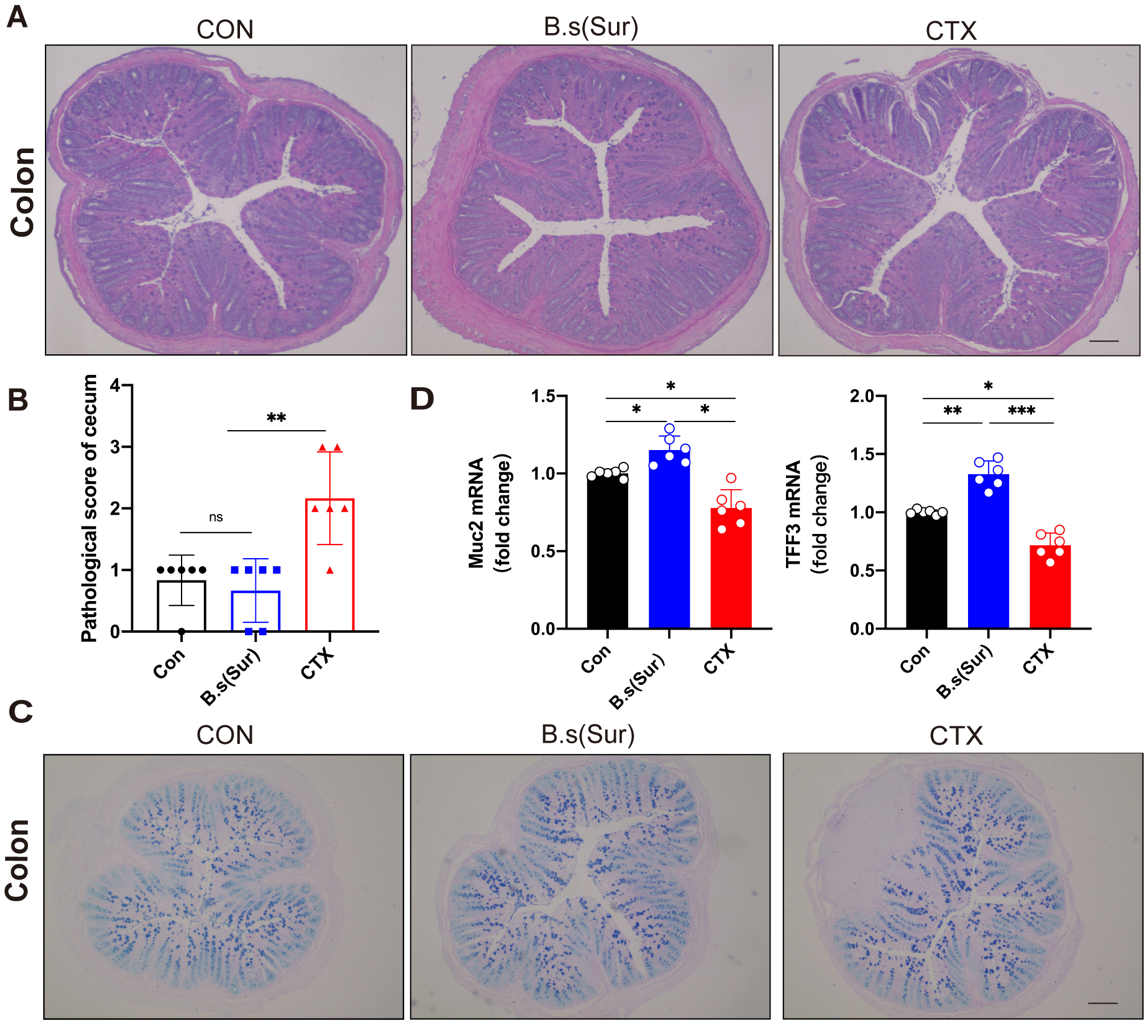
**

(A-D) Mice in the control group were given PBS orally for 21 days. The surfactin group was given orally 700 mg/kg surfactin for 21 days. The CTX group was given PBS orally for 21 days, followed by intraperitoneal injections of cyclophosphamide (100 mg/kg) on days 14-16. (A)Representative pictures of H&E staining in the Colon, Colon Scale bar 100 μm. (B) Histology score. (C) Representative pictures of Alcian blue staining in the Colon. Scale bar 100 μm. (D) qPCR analysis of the levels of *Muc2* and *TFF3* from Colon tissue. Data represent the mean ± SD of two or three independent experiments; comparisons performed with *t*-tests (two groups). **P* < 0.05, ***P* < 0.01, ****P* < 0.001, ns no statistical significance, *n* = 6 per group.

**Figure S2: The intestinal microbial diversity of surfactin pre-treated mice**


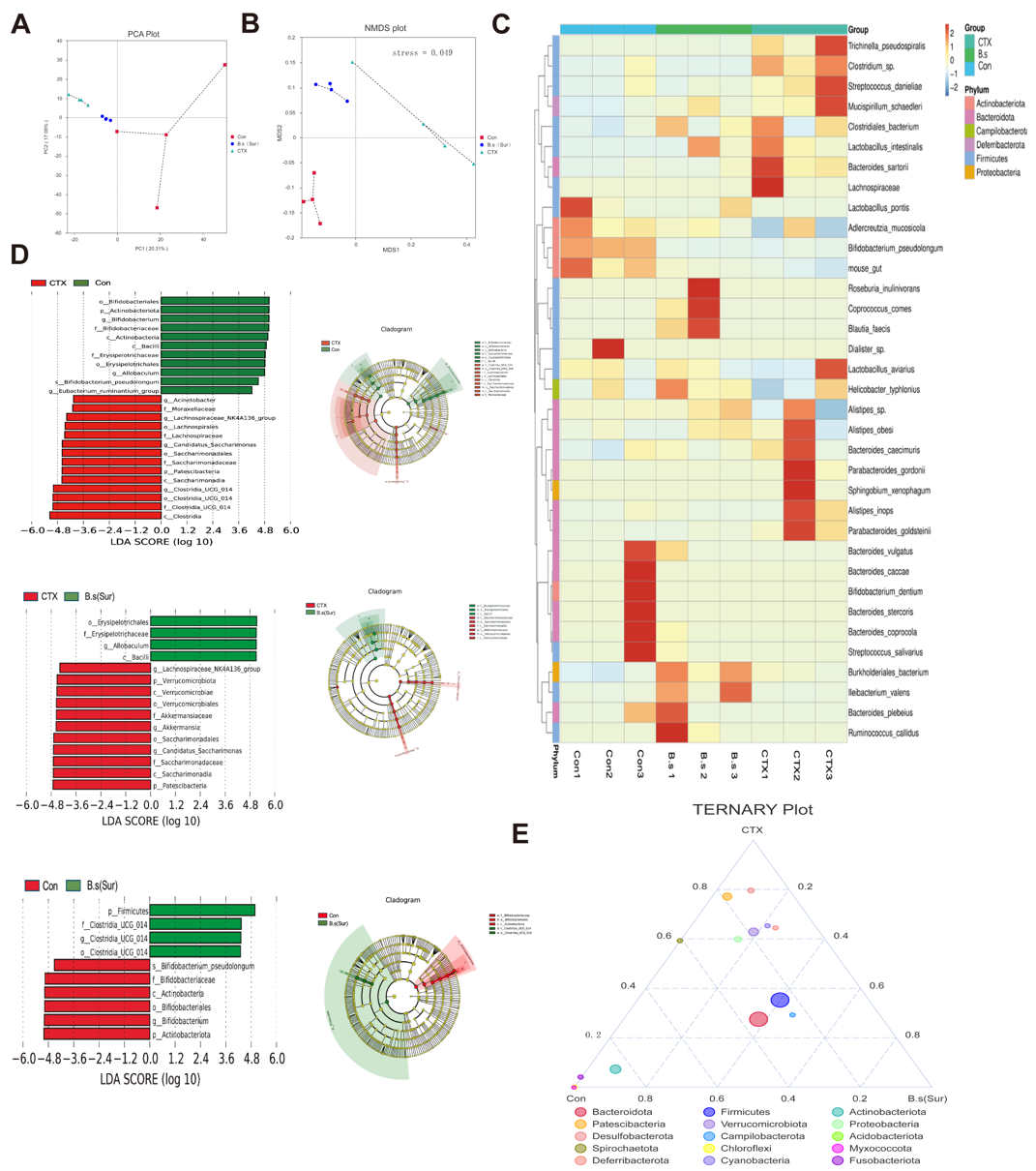


1. Two-dimensional PCA plots. (B）NMDS plots reflect the degree of between-sample and within-group variation through point-to-point distances (C) Cluster of species richness in different groups. (D) Analysis of differences in microbial taxa shown by linear discriminant analysis effect size. (E) Ternary Plot showing differences in dominant species among the three groups. *n* = 3 per group.

**Figure S3: Surfactin treatment ameliorated intestinal damage induced by CTX**


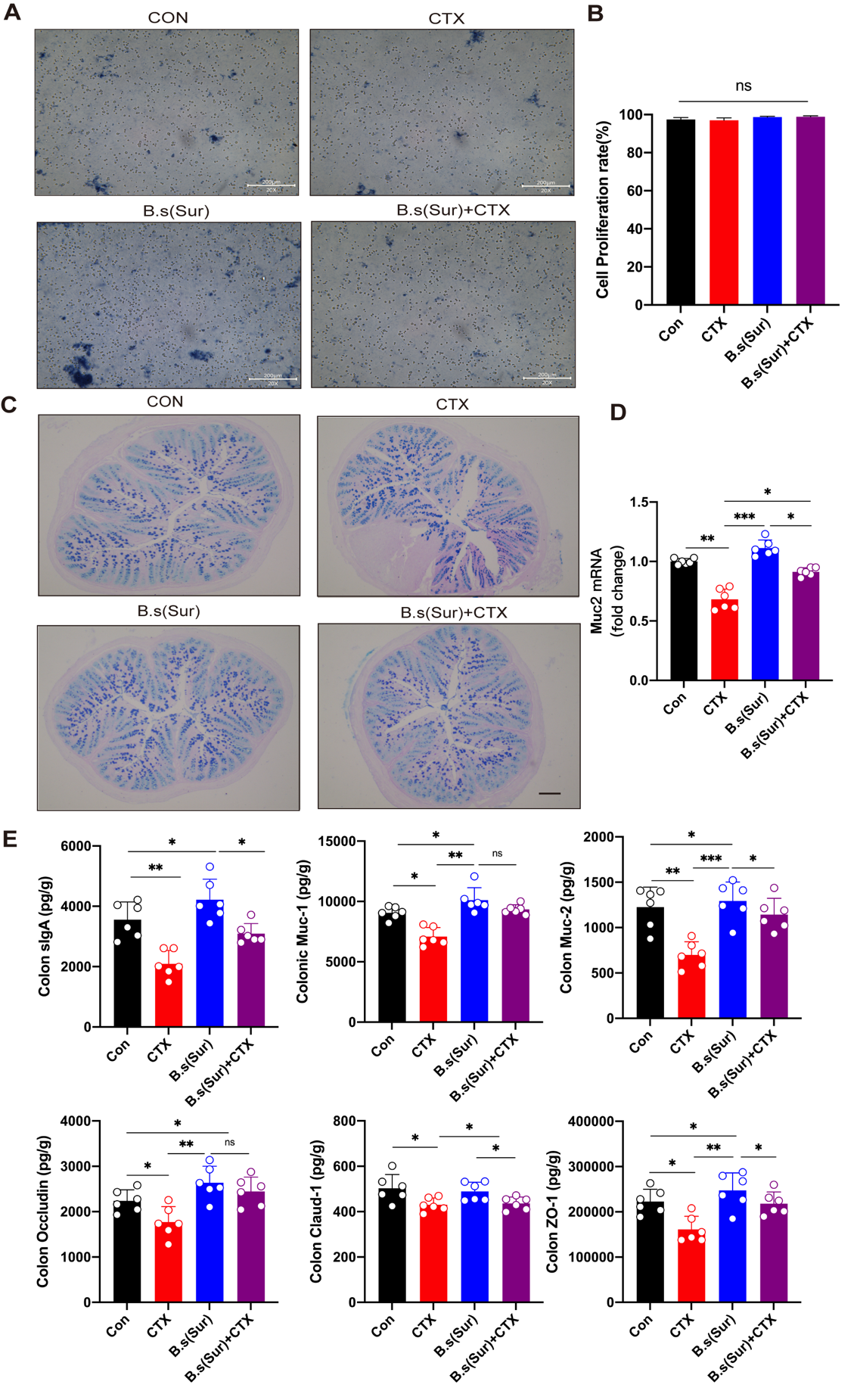


(A) Graph showing statistics on Taipan blue staining results. (B) Mouse spleen cells stained with Taipan blue staining. (C) Representative pictures of Alcian blue staining in the Colon. Scale bar100μm. (D) Graph showing qPCR analysis of the levels of Muc2 from Colon tissue. (E) ELISA of sIgA, Muc1, Muc2, occludin, claudin-1, and Zo-1expression in the Colon. Data represent the mean ± SD of two or three independent experiments; comparisons performed with *t*-tests (two groups). **P* < 0.05, ***P* < 0.01, ****P* < 0.001, ns no statistical significance, *n* = 6 per group.
